# Relationship of Iron Metabolism and Short-Term Cuprizone Treatment of C57BL/6 Mice

**DOI:** 10.3390/ijms20092257

**Published:** 2019-05-07

**Authors:** Edina Pandur, Ramóna Pap, Edit Varga, Gergely Jánosa, Sámuel Komoly, Judit Fórizs, Katalin Sipos

**Affiliations:** 1 Department of Pharmaceutical Biology, Faculty of Pharmacy, University of Pécs, H-7624 Pécs, Hungary; edina.pandur@aok.pte.hu (E.P.); pap.ramona@pte.hu (R.P.); edit.varga@aok.pte.hu (E.V.); janosa.gergely@gytk.pte.hu (G.J.); judit.forizs@aok.pte.hu (J.F.); 2 Department of Neurology, Medical School, University of Pécs, H-7623 Pécs, Hungary; komoly.samuel@pte.hu

**Keywords:** multiple sclerosis, cuprizone, iron metabolism, hepcidin, corpus callosum, liver

## Abstract

One of the models to investigate the distinct mechanisms contributing to neurodegeneration in multiple sclerosis is based on cuprizone (CZ) intoxication. CZ is toxic to mature oligodendrocytes and produces demyelination within the central nervous system but does not cause direct neuronal damage. The CZ model is suitable for better understanding the molecular mechanism of de- and remyelination processes of oligodendrocytes. CZ is a copper chelating agent and it also affects the iron metabolism in brain and liver tissues. To determine the early effect of CZ treatment on iron homeostasis regulation, cytosolic and mitochondrial iron storage, as well as some lipid metabolism genes, we investigated the expression of respective iron homeostasis and lipid metabolism genes of the corpus callosum (CC) and the liver after short-term CZ administration. In the present study C57BL/6 male mice aged four weeks were fed with standard rodent food premixed with 0.2 w/w% CZ for two or eight days. The major findings of our experiments are that short-term CZ treatment causes significant changes in iron metabolism regulation as well as in the expression of myelin and lipid synthesis-related genes, even before apparent demyelination occurs. Both in the CC and the liver the iron uptake, utilization and storage are modified, though not always the same way or to the same extent in the two organs. Understanding the role of iron in short-term and long-term CZ intoxication could provide a partial explanation of the discrepant signs of acute and chronic MS. These could contribute to understanding the development of multiple sclerosis and might provide a possible drug target.

## 1. Introduction

Multiple sclerosis (MS) is a disease of the central nervous system (CNS), causing inflammation and demyelination both in the grey and the white matter [1,2]. Several models have been developed to investigate the distinct mechanisms contributing to neurodegeneration in MS [3]. One of these models is based on cuprizone (CZ) intoxication [4]. CZ as a copper chelator, is toxic to mature oligodendrocytes and produces demyelination within the CNS, but does not cause direct neuronal damage [5,6]. Therefore, a CZ model is suitable for achieving a better understanding of the molecular mechanism of de- and remyelination processes of neurons. There are two effects attributed to CZ, the disturbance of copper homeostasis and the induction of copper chelate [7,8], but the exact mechanism of action of CZ is not fully understood. It is controversial whether in CZ treatment CZ can be reabsorbed from the duodenum and pass through the plasma membranes of neuronal cells [9]. Systematically, CZ treatment results in copper deficiency but does not affect the copper homeostasis of the brain. It has been proven that CZ disrupts energy metabolism of oligodendrocytes and triggers apoptosis [6,10], meanwhile the continuous administration of CZ leads to spontaneous remyelination [11]. The demyelination process due to CZ administration can be followed by monitoring the mRNA levels of IL-6 cytokine and growth/differentiation factor 15 (GDF15), a transcription factor expressed by oligodendrocytes in corpus callosum (CC) [12]. Activation of astrocytes showing a high expression level of glial fibrillary acidic protein (GFAP) is another sign of demyelination [13]. Astrocyte-microglia interactions with oligodendrocytes contribute to CZ-mediated de- and remyelination [14].

In CZ-affected brain regions, particularly in the corpus callosum, axonal injuries, microglia accumulation, and mitochondrial dysfunction can be observed [15,16]. The iron accumulation, the iron mediated mitochondrial dysfunction and ROS production contribute to cell death in neurodegenerative diseases [17,18,19]. Several heme-related disorders have been described in neurodegenerative diseases such as Alzheimer’s disease, Huntington’s disease, Friedreich’s ataxia, Parkinson’s disease, and multiple sclerosis. Hemoglobin-derived heme can have a neurotoxic effect in pathological conditions, as in free form it induces oxidative stress, lipid peroxidation, and inflammation [20]. Impairment of heme synthesis in the myelin sheath may contribute to demyelination during MS [21]. Moreover, the role of mutations in genes accounting for heme metabolism have been reported in neurodegenerative diseases [22,23]. Iron-sulfur cluster synthesis may also be affected by mitochondrial dysfunction in MS. NFS1 cysteine desulfurase is responsible for removing sulfur from cysteine, although its action depends on the iron content of the mitochondrion, therefore, iron deficiency or overload have a deep impact on iron-sulfur cluster biogenesis [24].

Several publications deal with the disturbances of iron homeostasis in multiple sclerosis [25,26,27,28]. Apotransferrin (aTf) secreted by oligodendrocytes is obligatory for glial differentiation while holotransferrin (Tf) transports iron, which is essential for myelin synthesis [29]. Intrathecal injection of Tf increased the mRNA as well as protein levels of myelin basic protein (MBP) and 2′,3′-cyclic-nucleotide 3′-phosphodiesterase (CNPase), without influencing those of proteolipid protein (PLP1) [30,31]. Tf via transferrin receptors also promotes the expression of genes related to mitochondrial function and lipid metabolism, necessary for myelin synthesis [32].

Since iron is necessary for normal brain functions and iron metabolism is connected to copper homeostasis, a large number of articles deal with the role of iron in MS [8,25,26,27,28,33]. It has been established that long-term (six weeks) cuprizone administration affected systemic iron metabolism by increasing liver iron content [33] and inducing megamitochondria formation [8]. Ferritin functions as an iron storage protein in the liver similarly to the function of microglia, neurons, and oligodendrocytes in the CNS [34]. Moreover, ferritin has an additional role in iron delivery in the white matter and in oligodendrocytes [35] since these CNS cells possess special ferritin receptors, which can also be found on hepatocytes [36]. In the cuprizone-induced demyelination model, ferritin expression increased [37] contributing to iron deposition and ameliorating iron-induced oxidative damage around the affected area [38]. Cuprizone treatment affects cytokine production of oligodendrocytes and astrocytes and activates microglia, which may have an effect on the brain and systemic iron homeostasis [39]. IL-6 is one of the strongest inflammatory stimuli produced by the cells mentioned earlier, which triggers the synthesis of hepcidin, the major regulator of iron metabolism [40]. Preprohepcidin is encoded by the HAMP (hepcidin antimicrobial peptide) gene. After the cleavage of the leader sequence prohepcidin protein is modified post-translationally by furin, a proprotein convertase enzyme [41]. The preprohepcidin-hepcidin maturation is regulated by alpha 1-antitrypsin (A1AT), a serine protease inhibitor that is able to bind prohepcidin and decrease its cleavage into mature hepcidin [42,43]. Hepcidin expression is regulated by many extracellular signals such as hypoxia, iron availability, and inflammation via different signaling mulecules (e.g., hemojuvelin (HJV), bone morfogenetic protein 6 (BMP6), TMPRSS6, hipoxia-inducible factor-1 (HIF1α), and transforming growth factor 15 (GDF15)) [41,44,45]. The interactions of these positive and negative transcriptional regulators maintains hepcidin synthesis [46,47,48].

Our previous experiments revealed that cuprizone as a copper chelating agent affected the iron metabolism in brain and liver tissues following four-week long cuprizone treatment [49]. To determine the early effect of CZ treatment on iron homeostasis regulation, cytosolic and mitochondrial iron storage, as well as some lipid metabolism genes we investigated the expression of respective iron homeostasis and lipid metabolism genes of the corpus callosum (CC) and the liver after short-term (two and eight days) CZ administration.

## 2. Results

### 2.1. Effect of CZ on the Expression of Activation Markers

**Figure 1:**
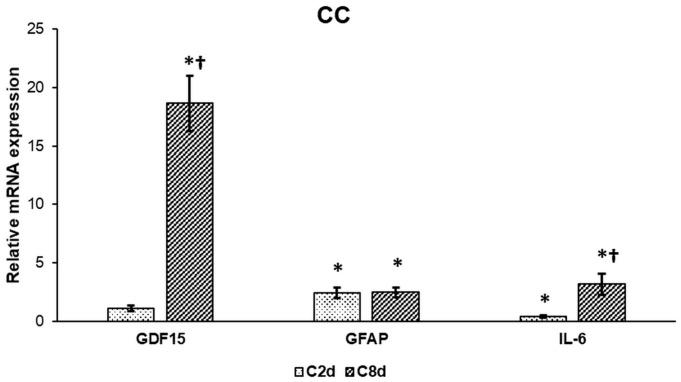
Relative mRNA expressions of GDF15, GFAP, and IL-6 in cuprizone (CZ) treated groups. Real Time PCR was performed with SYBR green protocol using gene specific primers. β-actin was used as housekeeping gene for the normalization and relative expression of control was considered as 1. The bars represent mean values and error bars represent standard errors of the mean (SEM) for three independent determinations. Student’s *t*-test was used to determine the statistical significances between the control and the treated groups. To determine the statistical significance between the treated groups a two-sample *t*-test was used. The asterisk shows *p* < 0.05 compared to the controls, the cross indicates difference *p* < 0.05 between the treated groups, *n* = 6 in each group.

To prove whether CZ administration activates glial cells in CC we examined the mRNA expression levels of the microglia activation markers IL-6 and growth/differentiation factor 15 (GDF15) and the astrocyte activation marker glial fibrillary acidic protein (GFAP). IL-6 and GDF15 mRNA expressions were elevated significantly after eight-day long CZ treatment suggesting microglial activation while GFAP expression significantly increased in both treated groups showing astrocyte activation (Figure 1).

### 2.2. Effect of CZ Administration on the Expression Levels of Hepcidin

**Figure 2:**
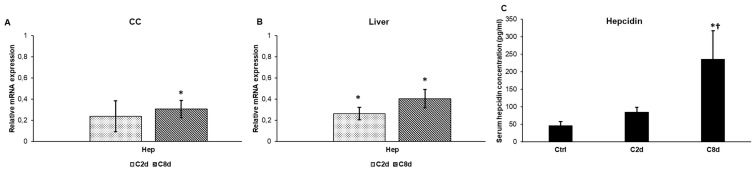
Relative hepcidin mRNA expression and serum hepcidin concentrations in non-treated and CZ-treated groups. Real Time PCR was performed with SYBR green protocol using gene specific primers. Relative expression of the control was regarded as 1. Hepcidin measurements were determined using mouse hepcidin-25 ELISA kit according to the manufacturer’s protocol. (**A**) Hepcidin mRNA expressions in corpus callosum (CC). (**B**) Hepcidin mRNA expressions in the liver. (**C**) Serum hepcidin concentrations. The bars represent mean values and error bars represent standard errors of the mean (SEM) for three independent determinations. For statistical analyses, Student’s *t*-test and two-sample *t*-tests were used. The asterisk shows *p* < 0.05 compared to the controls, the cross indicates the difference *p* < 0.05 between the treated groups, *n* = 6 in each group.

Hepcidin is responsible for the regulation of iron release mainly from hepatocytes and macrophages. In response to CZ administration, both in CC and in the liver preprohepcidin (HAMP) mRNA expressions showed decreased levels compared to the control group (Figure 2A,B).

Hepcidin, a liver-produced circulating hormone, is the major regulator of iron metabolism. To investigate the effect of CZ administration on hepcidin expression and release, hepcidin concentration was measured in serum samples of each animal groups—Ctrl, C2d, and C8d. Comparing the serum hepcidin concentrations of the treated groups to the values of Ctrl a slight increase of C2d was observed. In the C8d group, hepcidin concentration showed significant elevation compared to that in the control group (Figure 2C).

### 2.3. Effect of CZ Treatment on the Regulators of Hepcidin Expression

**Figure 3:**
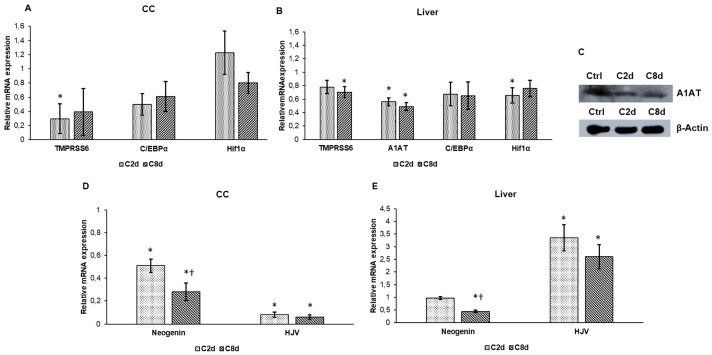
Expression analyses of hepcidin regulatory genes in CZ-treated groups. Real Time PCR was performed with SYBR green protocol using gene specific primers. Relative expression of controls was regarded as 1. Western blot analysis was done from protein extracts of the liver tissue samples. The same amount of protein (10 µg) from each sample was loaded onto 10% SDS-PAGE and transferred by electro blotting to nitrocellulose membranes and probed with A1AT polyclonal rabbit antibody according to the manufacturer’s protocol. β-actin was used as loading control. (**A**) Relative mRNA levels of the negative transcriptional regulator TMPRSS6 and the positive transcriptional regulators C/EBPα and HIF1α in CC. (**B**) Relative mRNA levels of TMPRSS6, C/EBPα, and HIF1α in the liver. (**C**) A1AT protein expression levels in the liver. The Western blot experiments were repeated at least three times in each group. (**D**) Relative mRNA levels of neogenin and HJV in the CC. (**E**) Relative mRNA levels of neogenin and HJV in the liver. The bars represent mean values and error bars represent standard errors of the mean (SEM) for three independent determinations. Student’s *t*-test was used to determine the statistical significances between control and treated groups. To determine the statistical significance between the treated groups a two-sample *t*-test was used. The asterisk marks *p* < 0.05 compared to non-treated animals, while the cross indicates *p* < 0.05 between the treated groups, *n* = 6 in each group. Optical density analysis can be seen in Figure S1.

After CZ administration the relative mRNA expression of matriptase-2 (TMPRSS6), a negative regulator of hepcidin expression, decreased both in CC and in the liver (Figure 3A,B) suggesting that downregulation of TMPRSS6 contributes to the upregulation of hepcidin secretion. We also examined the mRNA and protein levels of alpha 1-antitrypsin (A1AT), which regulates the prohepcidin cleavage by furin contributing to mature hepcidin production. Both mRNA and protein expressions of A1AT showed decreased levels in the liver (Figure 3B,C). C/EBPα and HIF1α the positive transcriptional regulators of HAMP did not show significant alterations in the mRNA expression levels compared to the controls both in the CC and in the liver (Figure 3A,B). We also examined the neogenin and hemojuvelin (HJV) expression levels which are involved in hepcidin regulation with TMPRSS6. mRNA expression of neogenin, which can facilitate the action of TMPRSS6, decreased in the liver, meanwhile the expression level of the positive regulator HJV markedly increased. In the CC the opposite change was found: HJV expression was downregulated (Figure 3D,E). CZ treatment significantly decreased neogenin expression levels in both treated groups compared to the control group (Figure 3D).

### 2.4. Effect of CZ Treatment on the Iron Storage and Transferrin Receptors Expressions

**Figure 4:**
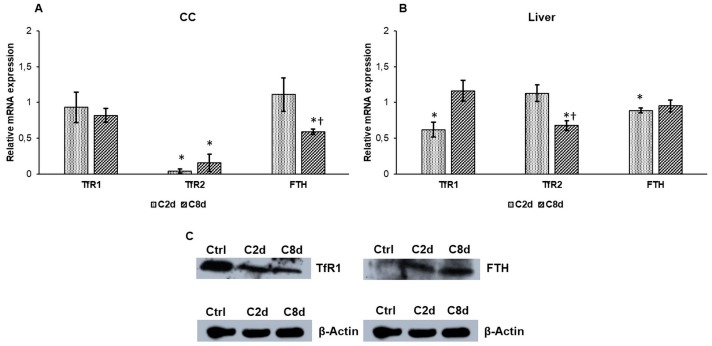
Relative mRNA expression values of transferrin receptor 1 (TfR1), transferrin receptor 2 (TfR2), and Ferritin heavy chain (FTH) and protein levels of TfR1 and FTH in CZ-treated groups. Real Time PCR was performed with SYBR green protocol using gene specific primers. β-actin was used as housekeeping gene for the normalization and relative expression of controls was regarded as 1. Western blot analyses were made from protein extracts of the liver tissue samples. Nitrocellulose membranes were probed with TfR1 and FTH polyclonal rabbit antibodies according to the manufacturer’s protocols. β-actin was used as loading control. (**A**) TfR1, TfR2, and FTH mRNA expressions in CC. (**B**) TfR1, TfR2, and FTH mRNA expression levels in the liver. (**C**) TfR1 and FTH protein expression levels in the liver. The Western blot experiments were repeated at least three times in each group. The bars represent mean values and error bars represent standard errors of the mean (SEM) for three independent determinations. For statistical analyses, Student’s *t*-test and two-sample *t*-tests were used. The asterisk shows *p* < 0.05 compared to the controls, while the cross indicates a difference *p* < 0.05 between the treated groups, *n* = 6 in each group. Optical density analyses can be seen in Figure S1.

Transferrin receptor 1 (TfR1) is responsible for the iron uptake into the cell, while transferrin receptor 2 (TfR2) serves as iron-sensing membrane protein. In the CC the expression level of TfR1 did not show a significant reduction in C2d and C8d (Figure 4A). In the liver TfR1 expression level decreased in C2d but it raised back to the control level in C8d (Figure 4B). Western blot analysis showed decreased TfR1 protein level in both tissues suggesting a delay between transcription and translation (Figure 4C). By comparison of TfR2, its relative mRNA expression was significantly reduced in the CC in both treated groups but in the liver its expression level showed relevant decrease only in C8d group. Ferritin heavy chain (FTH) was investigated to reveal the changes in the cytosolic iron storages in the CC and the liver. The gene expression analysis revealed significant decrease in the CC (Figure 4A). The FTH protein levels elevated at both time points of CZ treatment (Figure 4C).

### 2.5. Effect of CZ Administration on the Expressions of Mitochondrial Iron Transport and Storage Genes

**Figure 5:**
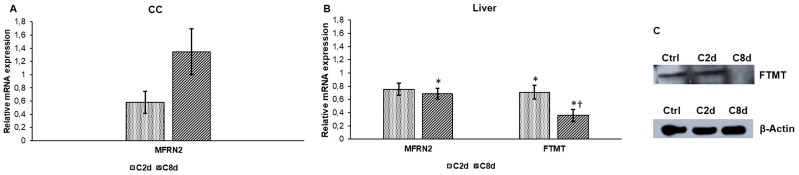
mRNA analyses of mitochondrial ferritin (FTMT) and mitoferrin-2 (MFRN2) genes in CC and in the liver and Western blot analysis of FTMT in the liver of CZ treated groups. Real Time PCR was performed with SYBR green protocol using gene specific primers. β-actin was used as housekeeping gene for the normalization and relative expression of controls was regarded as 1. Western blot analyses were made from protein extracts of the liver tissue samples. (**A**) Relative mRNA level of MFRN2 in CC. (**B**) Relative mRNA levels of FTMT and MFRN2 in the liver. (**C**) FTMT protein expression levels in the liver. The Western blot experiments were repeated at least three times in each group. The bars represent mean values and error bars represent standard errors of the mean (SEM) for three independent determinations. For statistical analyses, Student’s *t*-test and two-sample *t*-tests were used. The asterisk represents a difference *p* < 0.05 compared to non-treated animals, while the cross indicates *p* < 0.05 between the treated groups, *n* = 6 in each group. Optical density analysis can be seen in Figure S1.

Next, we examined whether CZ treatment influences mitochondrial iron transport (mitoferrin-2) and storage (mitochondrial ferritin) in CC and in the liver. The mitochondrial ferritin (FTMT) mRNA was not detected in the CC, but mitoferrin-2 (MFRN2) was measured with decreased expression value after two days and no significant rise was found after eight days (Figure 5A). As a result of CZ administration, the mRNA levels of MFRN2 and FTMT decreased significantly in the liver (Figure 5B). The downregulation of FTMT mRNA was seen at protein level as well in C8d (Figure 5C).

### 2.6. Expression Analysis of Mitochondrial Genes Involved in Heme and Iron-Sulfur Cluster Syntheses

**Figure 6:**
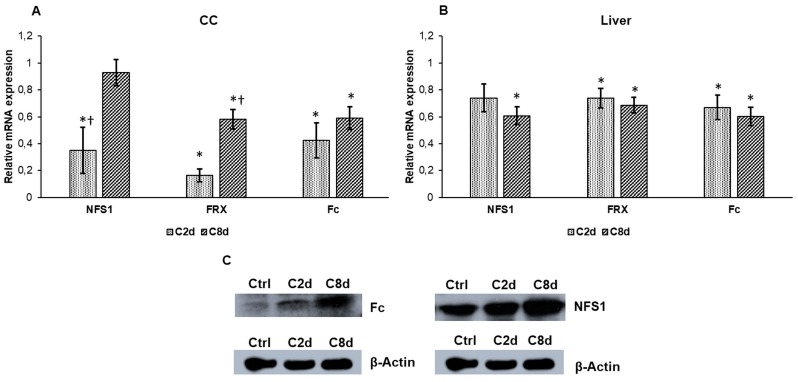
mRNA and protein analyses of NFS1, frataxin (FRX), and ferrochelatase (Fc) genes in CZ treated groups. Real Time PCR was performed with SYBR green protocol using gene specific primers. The relative expression of the controls was regarded as 1. Western blot analyses were done from protein extracts of the liver tissue samples. The same amount of protein (15 µg) from each sample was loaded onto 10% SDS-PAGE and transferred to nitrocellulose membranes and probed with NFS1 or Fc polyclonal rabbit antibodies. β-actin was used as loading control. (**A**) Relative mRNA expression values of NFS1, FRX, and Fc in CC. (**B**) Relative mRNA levels of NFS1, FRX, and Fc in the liver. (**C**) Protein expression levels of NFS1 and Fc in the liver. The Western blot experiments were repeated at least three times in each group. The bars represent mean values and error bars represent standard errors of the mean (SEM) for three independent measurements. For statistical analyses, Student’s *t*-test and two-sample *t*-tests were used. The asterisk marks *p* < 0.05 compared to non-treated animals, while the cross indicates a difference *p* < 0.05 between the treated groups, *n* = 6 in each group. Optical density analyses can be seen in Figure S1.

NFS1 cysteine desulfurase acts in the iron-sulfur cluster synthesis, showed decreased levels both in the CC (except in C8d group) and in the liver (Figure 6A,B), although its protein levels were increased in the liver, especially in the C8d group (Figure 6C). mRNA expression of frataxin (FRX) ferroxidase significantly decreased in the C2d group and it raised back close to the control level after eight-day-long CZ administration (Figure 6A). In the liver frataxin showed significant reduction compared to the control group (Figure 6B). Expression levels of ferrochelatase (Fc), the final enzyme of heme synthesis, were found to decrease compared to the controls both in the CC and in the liver (Figure 6A,B). Interestingly, Fc protein levels were elevated in the liver at both time points of the experiment (Figure 6C).

### 2.7. Heme and Non-Heme Iron Contents of the Liver Following CZ Treatment

**Figure 7:**
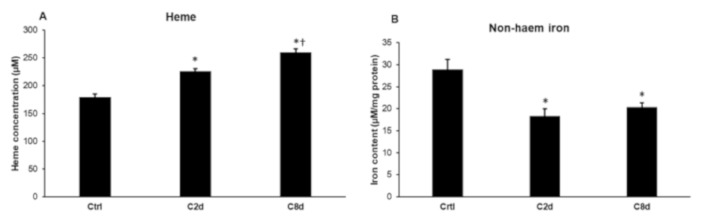
Heme concentrations and non-heme iron contents of the liver in non-treated and CZ-treated animals. Heme contents were determined using Heme Assay Kit according to the manufacturer’s protocol and were expressed in µM. Non-heme iron contents were determined using a colorimetric ferrozine-based assay. The values were expressed as µM iron/mg protein. (**A**) Heme concentrations of the liver samples of the CZ-treated animals. (**B**) Non-heme iron contents of the liver samples of the three animal groups. The columns represent mean values and error bars represent standard errors of the mean (SEM) for three independent measurements. For statistical analyses, a two-sample *t*-test was used. The asterisk marks *p* < 0.05 compared to non-treated animals, while the cross indicates a difference *p* < 0.05 between the treated groups, *n* = 6 in each group.

Based on the alterations of hepcidin synthesis and the expression of mitochondrial iron-related genes we assumed that heme content of the liver tissue may change on CZ treatment. We determined the heme concentration of the liver in each group of animals. The heme concentrations were significantly increased in both treated groups compared to the control group (Figure 7A). Next, we focused on the non-heme iron content of the liver samples. Since the heme concentrations were increased, we assumed that the non-heme iron would change as well. Non-heme iron content of the liver significantly decreased in response to CZ treatment both in C2d and C8d groups compared to the controls (Figure 7B).

### 2.8. Expression Analysis of Lipid Metabolism Genes Following CZ Treatment

**Figure 8:**
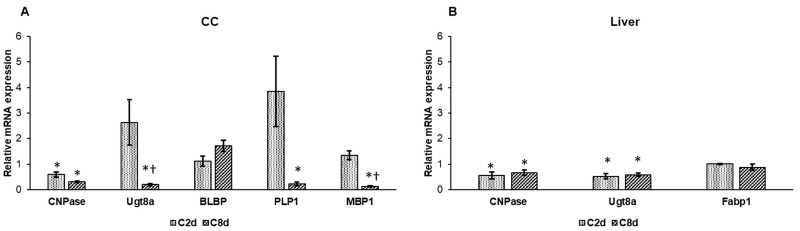
Relative mRNA expression levels of genes related to lipid metabolism. Real Time PCR was performed with SYBR green protocol using gene specific primers. β-actin was used as housekeeping gene for the normalization and relative expression of controls was considered as 1. (**A**) mRNA expression levels of 2′,3′-cyclic-nucleotide 3′-phosphodiesterase (CNPase), ceramide galactosyltransferase (Ugt8a), fatty acid-binding protein in brain (BLBP), proteolipid protein (PLP1), and myelin basic protein (MBP1) in CC. (**B**) Relative mRNA values of CNPase, Ugt8a, and fatty acid-binding protein (Fabp1) in the liver. The columns represent mean values and the error bars represent standard errors of the mean (SEM) for three independent measurements. Student’s *t*-test was used to determine the statistical significances between control and treated groups. To determine the statistical significance between the treated groups a two-sample *t*-test was used. The asterisk marks *p* < 0.05 compared to non-treated animals, while the cross indicates the difference *p* < 0.05 between the treated groups, *n* = 6 in each group.

To investigate the effect of CZ treatment on myelin sheath formation in the CNS we examined some of the genes that have been proven to have a role in myelin synthesis. The 2′,3′-cyclic-nucleotide 3′-phosphodiesterase (CNPase) mRNA expression was downregulated in both treated groups (Figure 8A). The ceramide galactosyltransferase (Ugt8a) mRNA expression was markedly elevated in C2d but after the eight-day long treatment it significantly decreased compared to the control group. The expression level of the fatty acid-binding protein in brain (BLBP), which is involved in hydrophobic ligand transports during CNS development, was increased (Figure 8A). Proteolipid protein (PLP1), which is the major myelin protein in the central nervous system, was highly upregulated in C2d, but it showed a significant reduction after eight-day long CZ treatment (Figure 8A). The mRNA expression of myelin basic protein (MBP1) was significantly decreased in the C8d samples (Figure 8A). To investigate the systemic effect of CZ on lipid metabolism, we determined the mRNA expression levels of CNPase, Ugt8a, and fatty acid-binding protein (Fabp1), which is responsible for cholesterol uptake and intracellular lipid transport in hepatocytes. CNPase and Ugt8a showed decreased expression rates compared to the control group, while Fabp1 expression did not change significantly due to CZ administration (Figure 8B).

## 3. Discussion

Multiple sclerosis (MS) can be characterized by progressive demyelination and axonal loss of the white matter of the central nervous system. A large number of animal models have been developed to characterize the clinical signs, molecular basis, diagnosis, and possible treatments of this chronic disease. One of these models is the mouse cuprizone (CZ) intoxication model, which mimics degeneration of mature oligodendrocytes [6]. CZ is a cupric chelating molecule; its precise mechanism of action is not fully understood. The classical CZ model means certain mouse strains treated with a defined (0.02%) concentration of CZ started at the appropriate age of the animal and lasted for 4–6 weeks [50]. This long-term treatment produces the morphological signs of demyelination in most of the cases accompanied by weight loss and characteristic movement disorders of the animals [7]. On cellular bases, the apoptosis of mature oligodendrocytes can be seen [10] together with microglia and astrocyte accumulation [51]. It seems necessary to administer CZ for at least three weeks for these obvious morphological signs to develop in the CNS of the experimental animals [52].

Experimental data are not unanimous as to whether CZ really binds cupric ion, and if so, what are those biochemical processes that are inhibited by this binding effect. Interestingly, after CZ treatment the copper level in the effected brain regions is not reduced but iron is accumulated in the liver [33] at the same time. Extra copper administration at the same time of CZ intoxication had no protective effect [9]. One significant consequence of CZ administration is the structural damage and production of megamitochondria of the liver as well as the brain [53].

In our previous works, we examined the effect of CZ on iron metabolism regulation both locally in the brain and systemically via the hormone hepcidin produced by the liver [49]. The reason for these experiments was that there is evidence that the metabolisms of the two ions (iron and copper) are closely related [54,55]. Furthermore, iron is involved in lipid and myelin synthesis [56]. Iron is released from the apoptotic oligodendrocytes and could be taken up by microglia and astrocytes [26]. The resulting regional iron accumulation could be observed in multiple sclerosis cases [57] and this can be associated with the presence of inflammatory signs and cause even further damage by provoking oxidative stress [57,58,59].

We found that after four weeks of CZ administration to mice both in the corpus callosum and in the liver significant iron metabolism regulation changes occurred. This meant that hepcidin and iron levels as well as iron utilization (especially iron-sulfur cluster synthesis) suffered dysregulation because of CZ intoxication.

In the present study, we wished to clarify whether short-term (two and eight days) CZ treatment causes any changes in iron metabolism of the CC and the liver, even before demyelination becomes obvious. We examined iron levels, hepcidin secretion and the expression levels of those proteins, which are involved in iron transport, storage, and utilization.

More and more experiments are dealing with the effects of short-term (less than two weeks) CZ treatment. The aim of these works is to gain a better understanding of the mechanism of the action of copper chelation and the relationship of this to demyelination. These could facilitate the development of multiple sclerosis; thus, a possible drug target might be found. Previous papers have proven that microglia cells and astrocytes are activated (with increased IL-6 secretion of the latter cell type) at one-week of CZ treatment even before demyelination happens [60]. Oligodendrocytes besides the myelin synthesis have a role in providing metabolites for energy generation of axons [61], and this process may be harmed by iron metabolism disturbance. Other changes after short-term treatment were apoptosis and downregulation of myelin synthesis genes of oligodendrocytes, which could be seen as early as after six days of CZ intoxication [62]. Goldberg and co-workers have found that after four days of CZ administration to mice, expression of genes responsible for myelin synthesis in the corpus callosum were reduced [63]. Additionally, they found amino acid metabolism disturbances in the liver, which proved that CZ has a local effect in the brain as well as a systemic effect on liver metabolism.

It looks obvious that the damage of mitochondria is an early sign of the CZ mouse multiple sclerosis model [8]. Proper functioning of mitochondria is needed for meeting the huge energy demand of the cells of the CNS for myelin and neurotransmitter synthesis as well as for providing the necessary ion movement. Iron is required for all the functions of the brain mentioned above. Mitochondria play a crucial part in cellular iron metabolism, as iron-sulfur cluster biogenesis, the final step of heme synthesis, and terminal oxidation also take place in mitochondria [64]. Many malfunctions of iron metabolism regulation can lead to intracellular and mainly intra-mitochondrial iron accumulation, which can be the basis for generation of oxidative stress [59].

We have administered CZ to mice for two and eight days, after which time they were sacrificed and analyzed to examine the iron metabolism of the corpus callosum and the liver. In this time interval no demyelination occurred, but multiple metabolic changes could be observed by other research teams. Lately, Scheld et al. proved that after two days of CZ treatment a large IL-6 secretion could be seen by oligodendrocytes in the corpus callosum of the animals [12]. The same elevation of IL-6 expression was noticed in stressed oligodendrocytes, which was accompanied by a similarly high GDF15 mRNA level. The transcription factor GDF15 level is found elevated mainly in patients with ineffective erythropoiesis (e.g., thalassemia) [65] causing hepcidin level downregulation in these cases.

Hepcidin is the main hormone regulating iron level in the blood via ferroportin, an iron exporter [66]. Hepcidin expression occurs mainly in the liver, and is under complex regulation by iron level, hypoxia, and inflammation [67]. In our present experiments, the expression of HAMP coding for preprohepcidin was reduced significantly in the liver as well as in the CC after eight days of CZ treatment. This seems controversial to the elevation of the serum level of the mature hepcidin peptide. When we examined the changes of the regulators of HAMP expression and hepcidin maturation, we found that both the mRNA and the protein levels of A1AT in the liver decreased. As this protein is responsible partially for prohepcidin-hepcidin conversion [42], this can explain the relative increase of mature hepcidin level in the blood. There are only a few negative regulators of preprohepcidin mRNA synthesis, they are responsible for providing iron in the case of hypoxia and in need for erythropoiesis [67]. The latter factors include GDF15, the level of which increased enormously after eight days of CZ administration. In the CC of treated mice, the level of the negative hepcidin regulator matripase-2 (TMPRSS6) did not change significantly, but neogenin and hemojuvelin expressions decreased at both time points of the experiment. Neogenin is also a receptor for repulsive guidance molecule A (RGMa) in the brain, they are important in the regeneration processes and angiogenesis of the brain [68]. Interestingly, in the liver neogenin mRNA level also decreased but HJV expression was elevated after CZ treatment. As the presence of neogenin is essential for HJV to activate BMP/SMAD pathway for maintaining HAMP expression in the liver [69], the elevation of HJV expression could be considered as a compensatory attempt of the hepatocytes. All these mRNA level changes may serve as an explanation for the reduced expression of the HAMP gene both in the CC and in the liver.

If we compare the changes of hepcidin and iron levels between long-term (four weeks) and short-term (two and eight days) CZ treatments, we can observe that serum iron levels are not altered after a few days’ treatment (data not shown) but are reduced significantly after weeks. The mRNA levels of preprohepcidin were modified opposingly in the two treatments while the serum levels of hepcidin increased in both cases, but only in the short time interval was the elevation significant.

These facts seem to prove that CZ administration has a very fast response concerning HAMP expression, but these few days are not long enough to see definite changes in serum iron concentration. It seems obvious that the steps the liver and the CC take in response to CZ intoxication are different in short- and long-time intervals, so the “acute” and “chronic” regulators are not the same.

Considering the alterations occurring in cytosolic iron uptake and storage in CC of CZ treated mice, the most prominent result was the extreme reduction of TfR2 mRNA level after two days. This receptor together with HFE is rather a sensor of blood iron level [70] and regulates hepcidin expression. Heidari et al. [71] describe a striking effect of double mutation of HFE/TfR2 in mice: In these animals significant iron accumulation developed in the white matter of the brain, though of the iron related genes only ceruloplasmin and FTL expressions were modified. They found that the mRNA of a large number of myelin related genes were reduced in mutated mice. As the relationship of iron metabolism and myelin synthesis is well known [56], it is intriguing that this regulatory loop is activated after just two days of CZ administration. The level of mRNAs of lipid related genes decreased dramatically after eight days of CZ treatment and the same alterations were observed after long-term (four weeks) treatment. Lipid synthesis was diminished similarly in the liver as well, proving a systematic effect of copper chelation and iron metabolism disturbance.

Mitochondria are affected in many ways in MS. Myelin synthesis requires constant and increased ATP production. The appearance of megamitochondria suggests energy deficit in oligodendrocytes and hepatocytes as one of the consequences of copper chelation. Apart from the malfunction of iron metabolism, regulation causes mitochondrial iron accumulation, which is a source of oxidative stress [58]. After long-term CZ administration we could see mitochondrial iron uptake (MFRN2) and incorporation (NFS1, Fc, FRX) reduction only in the liver but not in the CC of mice. Contrary to the long-term CZ administration, after short-term CZ treatment the expression of genes involved in mitochondrial iron uptake and utilization were decreased not only in the liver, but the latter ones in the CC as well. The tendency of the change is different in the two organs: The mRNA levels of the genes responsible for iron-sulfur cluster and heme syntheses drop early (two days) in CC and elevate later, but in the liver the reduction of expressions continues with time. The non-heme iron content of the liver diminished rapidly and remained low after four weeks, while the heme-iron level increased in a short time interval. This may mean that the liver redistributes the iron containing products according to the need of the extrahepatic tissues.

In summary, we can conclude that short-term CZ treatment causes a definite and significant change in iron metabolism regulation together with the expression of myelin and lipid synthesis related genes before apparent demyelination. This change is obvious in the CC as well as in the liver, so it is a local and a systemic effect, at the same time. The iron uptake, utilization, and storage are not always modified the same way in the two organs, which may suggest a difference in the regulation of iron metabolism in the CNS and systematically via the liver. As functional consequences, we may conclude that the serum level of the major iron metabolism regulatory hormone increased, which caused further very rapid iron storage and utilization outcome. The expression levels of genes responsible for incorporating iron into heme and iron-sulfur clusters have been reduced, especially in the liver after a few days of CZ administration. The heme content of the liver increased, which could mean a protective response. All of these iron metabolism changes have a major effect on the energy production of mitochondria, both in liver and in CNS. As a secondary answer to the iron metabolism disturbance in the CC, the lipid and myelin syntheses seemed to be withheld, which is a significant sign of the beginning of demyelination. Interestingly the lipid metabolism is also damaged in the liver, which indicates that CZ intoxication has a systematic metabolic effect. The behavior of iron could be distinct in short-term and long-term CZ intoxication, which could be a partial explanation to the variations of the signs of “acute” and “chronic” MS.

## 4. Materials and Methods

### 4.1. Animals

C57BL/6 male mice aged four weeks were separated into three groups with six animals in each. The control group (Ctrl) received normal food for eight days. The treatment groups were fed with standard rodent food premixed with 0.2 w/w% CZ (Sigma-Aldrich Kft., Budapest, Hungary). The first treatment group (C2d) were kept on CZ diet for two days while the second one (C8d) were fed for eight days. Right before and at the end of the treatment blood was drawn from each mouse for serum measurements. Animals were kept under SPF conditions under permits BAI/01/1390-003/2013 (issued by the Baranya County Government Office) and SF/688-18/2013 and SF/27-1/2014 (issued by the Ministry of Agriculture). The Institutional Animal Care and Use committee has approved the animal use protocols (SF/688-18/2013 approved date: 14 February 2013 and SF/27-1/2014 approved date: 19 February 2014). All applicable international, national, and/or institutional guidelines for the care and use of animals were followed.

### 4.2. RNA Isolation and Real Time PCR

For gene expression analysis, mice were anesthetized with urethane and were sacrificed right after the respective treatment time. Brains and the livers were removed, frozen in dry ice and stored at −80 °C for further use. For the preparation of the corpus callosum (CC) the frozen brains were thawed, segmented in coronal and sagittal planes. The diencephalon as well as the cortex were removed, and the CCs were collected. In the liver, after thawing, sections of the same weight were sliced. For total RNA isolation the Zymo Quick-RNA Miniprep Kit (Zymo Research, Irvine, CA, USA) was used for both tissues. Equal amount (100 ng) of total RNA was reverse transcribed to cDNA by the High Capacity cDNA Reverse Transcription Kit (Applied Biosystems, Waltham, MA, USA). Quantitative Real Time PCR was run for the genes of interest with the CFX96 Touch Real Time PCR Detection System (Bio-Rad Incorporation, Hercules, CA, USA) using iTaq Universal SYBR Green Supermix (Bio-Rad Inc.). Results were analyzed with the Bio-Rad CFX Manager Software Version 3.1 (Bio-Rad Inc.) using the comparative 2^−∆∆*C*t^ (Livak) method. In the analysis of gene expression, the results of non-treated animals (Ctrl) were taken as a reference level with a value of 1. β-actin was used as housekeeping gene for the normalization of the expression levels of the target genes. The primer sequences used in this study are described in Table S1.

### 4.3. Serum Hepcidin Measurements

Blood samples were processed as a serum of the six animals from each group. Blood was drawn at the end of the second and eighth days of CZ treatments. Control blood samples were collected from the non-treated animals after eight days. Serum hepcidin concentrations were determined with sandwich enzyme linked immunosorbent assay (ELISA) specific for mouse hepcidin-25 (Abbexa Ltd., Cambridge, UK) according to the manufacturer’s protocol. The measurement was carried out at 450 nm using MultiSkan GO spectrophotometer (Thermo-Fischer Scientific Inc., Waltham, MA, USA).

### 4.4. Non-Heme Iron Measurements

Non-heme iron contents of the liver samples were determined according to the protocol of Rebouche et al. [72] with the following modification: The samples were measured with a microplate in a MultiSkan GO spectrophotometer (Thermo-Fischer Scientific Inc.). Standard curves were obtained using FeCl_3_ standards treated the same way as the samples. The protein contents of the liver were defined from the supernatant of the lysed tissues with DC protein assay kit (Bio-Rad Inc.) where bovine serum albumin was used as the standard. The iron contents of the liver samples were calculated as µM/mg of protein content of the supernatants.

### 4.5. Heme Measurements

Total heme concentrations of the liver samples were measured with Heme Assay Kit (Sigma-Aldrich Kft., Budapest, Hungary) according to the manufacturer’s protocol with the following modification: 50 mg of each sample were homogenized in 500 µL of ultrapure water using potter homogenizer. Heme concentrations of the liver samples were expressed as µM.

### 4.6. Western Blot Analysis

The liver tissues (100 mg) were homogenized in 500 µL of RIPA buffer [25 mM Tris–HCl pH 7.6, 150 mM NaCl, 1% NP-40, 1% sodium deoxycholate, 0.1% SDS, Complete Mini protease inhibitor cocktail (Roche Diagnostics, Meylan, France)] with Dounce homogenizer. The homogenates were clarified by centrifugation at 12,000 rpm for 10 min at 4 °C. The protein contents of the supernatants were measured with DC Protein Assay Kit (Bio-Rad Inc.). The same amount of protein from each sample was loaded onto 12% or 14% SDS-PAGE and transferred to nitrocellulose membranes (BioTrace NT, Pall Life Sciences, Port Washington, NY) and probed with rabbit polyclonal antibodies produced against mouse ferrochelatase (Fc 1:1000; Novus Biologicals), mitochondrial ferritin (FTMT 1:1000; Thermo-Fischer Scientific Inc.), ferritin heavy chain (FTH 1:1000; Cell Signaling Technology Europe, Leiden, The Netherlands), NFS1 (1:1000; Novus Biologicals), transferrin receptor-1 (TfR1 1:1000; Thermo-Fischer Scientific Inc.) and alpha-1 antitrypsin (A1AT 1:1000; Abcam, Cambridge, UK). β-actin (1:2000; Sigma-Aldrich Kft.) was used as loading control. Goat anti-rabbit (H + L) HRP-conjugate was used as secondary antibody (1:3000; Bio-Rad Inc.). Protein detection was carried out with WesternBright ECL chemiluminescent substrate (Advansta Inc., San Jose, CA). All experiments were repeated at least three times. Optical densities of the Western blots were calculated by ImageJ software (https://imagej.nih.gov/ij) and expressed as percentage of target gene/β-actin abundance.

### 4.7. Statistical Analysis

Data were obtained from six independent experiments. Results are expressed as mean ± standard error of means (SEM). Statistical analysis was performed using SPSS software (IBM Corporation, Armonk, NY, USA). Statistical significance was determined using a Student’s *t*-test to compare control group to treated groups (Ctrl vs. C2d and Ctrl vs. C8d) and a two-sample *t*-test to compare the two treated groups. The *p* value < 0.05 was determined statistically significant difference.

## Supplementary Materials

Supplementary materials can be found at https://www.mdpi.com/1422-0067/20/9/2257/s1.
