# Supplementary material for: Relationship of Iron Metabolism and Short-Term Cuprizone Treatment of C57BL/6 Mice
Source: Int J Mol Sci. 2019 May 7;20(9):2257. doi: 10.3390/ijms20092257 (PMC6539941; doi:10.3390/ijms20092257)
Supplement: Supplementary file 1 [file ijms-20-02257-s001.zip › Supplementary files/Supplementary material.docx]

**Supplementary material**

**Figure S1:** Optical densities of the western blot analyses of A1AT (**A**), TfR1 (**B**), FTH (**C**), FTMT (**D**), Fc (**E**) and NFS1 (**F**) of Ctrl and CZ treated animal groups. The analyses were made using ImageJ software (https://imagej.nih.gov/ij/), the optical density of the examined proteins was expressed as percentage of target protein/β-Actin abundance. Asterisk marks p < 0.01 compared to untreated controls.
