# Supplementary material for: Relationship of Iron Metabolism and Short-Term Cuprizone Treatment of C57BL/6 Mice
Source: Int J Mol Sci. 2019 May 7;20(9):2257. doi: 10.3390/ijms20092257 (PMC6539941; doi:10.3390/ijms20092257)
Supplement: Supplementary file 1 [file ijms-20-02257-s001.zip › Supplementary files/Supplementary Table 1.docx]

**Supplementary Table 1.** **Real-time PCR gene primer list**

| **Primer** |  | **Sequence 5'→ 3'** |
| --- | --- | --- |
| β-actin | forward | CTGTCGAGTCGCGTCCA |
|  | reverse | TCATCCATGGCGAACTGGTG |
| Hepcidin1 | forward | GACATTGCGATACCAATGCAG |
|  | reverse | GCAACAGATACCACACTGGGA |
| GDF15 | forward | CGCTCCAGGCCCAGC |
|  | reverse | GGGTCGCTGTTCAGGCATT |
| GFAP | forward | CAGATCCGAGGGGGCAAA |
|  | reverse | GGTCAGGGCTCCATTTTCAATC |
| IL-6 | forward | CTCTGCAAGAGACTTCCATCCA |
|  | reverse | GACAGGTCTGTTGGGAGTGG |
| TMPRSS6 | forward | AACCTGTGATGGAGGTGCTG |
|  | reverse | TGGGGTAGTAGGGTGTACGG |
| A1AT | forward | TCTGGGACAGCAAGCTGAAA |
|  | reverse | GCTGGGGACTGATCCTTCTG |
| C/EBPα | forward | GCAAAGCCAAGAAGTCGGTG |
|  | reverse | GCGGTCATTGTCACTGGTCA |
| HIF1α | forward | GCCTTAACCTGTCTGCCACT |
|  | reverse | GCTGCTTGAAAAAGGGAGCC |
| HJV | forward | TGCCAGAAGGCTGTGTAAGG |
|  | reverse | AAGATTCGGGCATCGTCCAG |
| Neogenin | forward | CAGCCCGCAGTCATCTCTAC |
|  | reverse | TGTGGAATTGGCCCTGTCTG |
| TfR1 | forward | TCCGCTCGTGGAGACTACTT |
|  | reverse | ACATAGGGCGACAGGAAGTG |
| TfR2 | forward | GGTCCTGATCACCCTGCTAA |
|  | reverse | GGAGGTCGCTCCAGTACAAC |
| FTH | forward | GACCGAGATGATGTGGCTCT |
|  | reverse | GTGCACACTCCATTGCATTC |
| MFRN2 | forward | CCACTGTCACCACGCACAT |
|  | reverse | GCCTCCAACACGTTCCGATA |
| FTMT | forward | GAGCTTGCCTGACCTCAGTT |
|  | reverse | AGTGCAGAGCTGATGTGCTT |
| NFS1 | forward | GATTGGAGCTGATCCTCGGG |
|  | reverse | AGAACCTGGCCACTCCCTTA |
| FRX | forward | GGGAACCGATCGTAACCTGC |
|  | reverse | AGTGGAGGTTCAAATGGGCG |
| Fc | forward | ACCACAGGCAGCAGCTTAAA |
|  | reverse | CCTGTCGATTGTGCTCCACT |
| CNPase | forward | GTGTGCTGCACTGTACAACC |
|  | reverse | CTCTTCACCACCTCCTGCTG |
| Ugt8a | forward | GCCGAAGGACGCGCTAT |
|  | reverse | TCAAACATAATTGGCGGCACA |
| BLBP | forward | CTGTGGTTCGGTTGGATGGA |
|  | reverse | TCCCCAAAGGTAAGAGTCACG |
| PLP1 | forward | CTTCCTTTATGGGGCCCTCC |
|  | reverse | GTGATGCCCACAAACGTTGC |
| MBP1 | forward | AGAAGAGACCCTCACAGCGA |
|  | reverse | CTAAAGAAGCGCCCGATGGA |
