# Supplementary figures and images for: Relationship of Iron Metabolism and Short-Term Cuprizone Treatment of C57BL/6 Mice
Source: Int J Mol Sci. 2019 May 7;20(9):2257. doi: 10.3390/ijms20092257 (PMC6539941; doi:10.3390/ijms20092257)

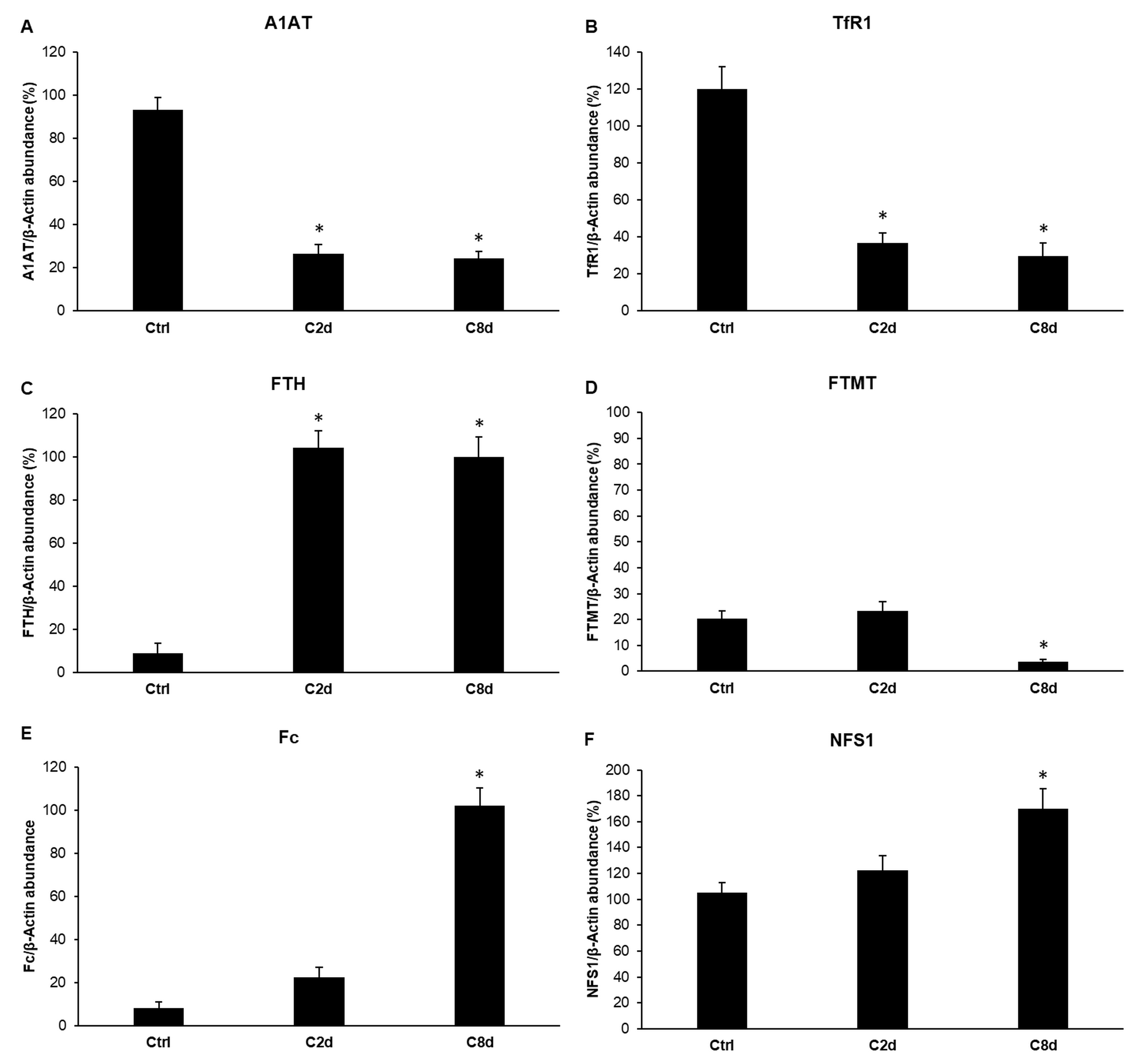

Supplement: Supplementary file 1 [file ijms-20-02257-s001.zip › Supplementary files/Figure S1.tiff]
